# The complete mitochondrial genome of the Antarctic marine triclad, *Obrimoposthia Wandeli* (Platyhelminthes, Tricladida, Maricola)

**DOI:** 10.1080/23802359.2019.1640093

**Published:** 2019-07-13

**Authors:** Hee-Min Yang, Su-Jung Ji, Gi-Sik Min

**Affiliations:** Department of Biological Sciences, Inha University, Incheon, South Korea

**Keywords:** Mitogenome, Platyhelminthes, marine Tricladida, *Obrimoposthia wandeli*

## Abstract

For the first time, we report the complete mitochondrial genome (mitogenome) sequence of the marine triclad species, *Obrimoposthia wandeli*. The complete mitogenome of *O. wandeli* was 15,185 bp in length, contains 12 protein-coding genes (PCGs), 22 transfer RNAs (tRNAs), and 2 ribosomal RNAs (rRNAs). Compared to previously reported gene orders from the order Tricladida, *O. wandeli* had unique gene order. We constructed a phylogenetic tree based on the mitogenomes belonging to Rhabdocoela, Polycladida, and Tricladida and confirmed that *O. wandeli* is located in the basal position in the Tricladida.

The order Tricladida Lang, 1884, often called planarians, contains more than 1700 species. Despite the numerous species in the Tricladida, only seven complete or nearly complete mitogenome sequences have been reported (Sakai and Sakaizumi 2012; Solà et al 2015; Ross et al. 2016). Of these seven species, five are freshwater triclads and two are land triclads. In this study, we determined the complete mitogenome of *Obrimoposthia wandeli* (Hallez 1906) and this is the first report of a complete mitogenome sequence for a marine triclad species belonging to the suborder Maricola Hallez, 1892, a small triclads group with approximately 80 species (Sluys 1989a).

Specimens of *Obrimoposthia wandeli* were collected from a pebble beach near the King Sejong Station, King George Island in Antarctica (62°12′S, 58°47′W). Collected flatworms were transferred live to the laboratory and starved for a week to ensure that DNA from other organisms was not extracted during the DNA extraction step. Mitochondrial DNA extraction, sequencing, and gene annotation were performed using the methods described by Song et al. (2016). The extracted mitochondrial DNA was deposited in the DNA collection at the National Institute of Biological Resources, Incheon, South Korea (deposit no. NIBRGR0000603781). For phylogenetic analysis, we used 1, 2 codon positions of 11 protein-coding gene (PCG) sequences. Selection of PCGs and 1, 2 codon positions was conducted using DAMBE 7.0.35 (Xia 2017). The maximum-likelihood tree was constructed using IQ-tree 1.6.10 with the GTR + F+I + G4 model (Kalyaanamoorthy et al. 2017; Hoang et al. 2017).

The complete mitogenome of *O. wandeli* (GenBank accession number: MK962607) was 15,185 bp in length and had 12 PCGs, 22 tRNAs, and 2 rRNAs. Like other mitogenomes of the Platyhelminthes species, *O. wandeli* did not have *ATP8* gene.

In the previous studies, six types of PCG orders were reported in the phylum Platyhelminthes (Catenulida, Macrostomida, Rhabdocoela, Polycladida, Tricladida, and a parasitic group). Until now, seven complete or nearly complete mitogenomes have been recorded in the order Tricladida and all mitogenomes had the same PCG arrangement. The *O. wandeli* shared some common features with other known triclads in the PCG arrangement. *ND6*-*ND5* genes, *ND4L*-*ND4* genes, and *CO2*-*ND3*-*ND2* genes were arranged similarly. Especially, *CO2*-*ND3*-*ND2* gene order was only found in the Tricladida type gene arrangement. However, except for these three gene grouping, there were many differences in the PCG arrangement of *O. wandeli* in comparison with those of other triclads.

To confirm a phylogenetic relationship, we constructed a maximum-likelihood tree based on the 11 PCG concatenated sequences from 10 species mitogenomes belonging to the Rhabdocoela, Polycladida, and Tricladida. Polycladida was used as an outgroup. The *O. wandeli* was grouped with other Tricladida species and formed a monophyly (Figure 1). The basal position of *O. wandeli* in the Tricladida was congruent with the previous phylogenetic study (Sluys 1989b; Carranza et al. 1998; Riutort et al. 2012). However, because we found a new gene order in the *O. wandeli*, we need to obtain more mitogenome data of marine triclads to clarify the gene order and phylogenetic position of the suborder Maricola.

**Figure. 1.**
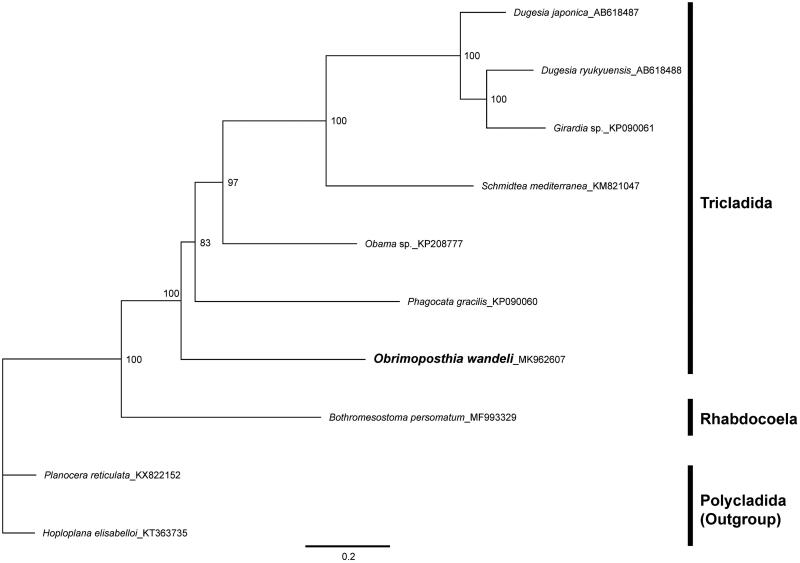
Maximum-likelihood (ML) tree based on the mitogenome sequences of *O. wandeli* and Polycladida, Rhabdocoela, Tricladida species. The bootstrap supports are shown on each node.
